# Management of an intraoperative tracheal injury during a Mckeown oesophagectomy: A case report

**DOI:** 10.1016/j.ijscr.2023.108010

**Published:** 2023-03-21

**Authors:** B.M. Munasinghe, C.T. Karunatileke

**Affiliations:** a Department of Anaesthesiology and Intensive Care, Queen Elizabeth the Queen Mother Hospital, Margate, Kent, UK; b Department of Surgery, District General Hospital, Mannar, Sri Lanka

**Keywords:** Tracheobronchial injury, Airway injury, Tracheal, Primary repair, Mckeown oesophagectomy, Case report

## Abstract

**Introduction and importance:**

Tracheobronchial injuries are uncommon complications during oesophagectomies adopting blind dissection or thoracoscopy. Neoadjuvant chemo-radiotherapy is considered a risk factor while double-lumen endotracheal tube insertion and direct surgical damage are other related causalities.

**Presentation of case:**

A 65-year-old male underwent a Mckeown oesophagectomy with a right thoracotomy for a mid-oesophageal carcinoma. During the latter stages of cervical dissection and oesophageal mobilization, a 2-cm tracheal injury was noted in the posterior membranous trachea. It was repaired with 2.0 prolene with interrupted sutures and local transposition muscle flap using prevertebral muscles. Post-operatively, he was ventilated in view of prolonged surgery and the probability of airway oedema with the double-lumen ET tube. A transient bubbling of the intercostal drain was managed conservatively and attributed to a secondary pneumothorax. He was extubated and made an uncomplicated recovery. At 2 years, he did not have any tracheal stenosis.

**Clinical discussion:**

If diagnosed intraoperatively and for sizes >2 cm, tracheobronchial injuries should be repaired. Various techniques exist with differing evidence. Repair with non-absorbable sutures, use of synthetic grafts, innate tissue such as intercostal and pectoral muscle flaps, and pericardial and pleural flaps are all being used. Early extubation might be useful provided other criteria for extubation are met.

**Conclusion:**

Tracheobronchial injuries during oesophagectomies present a surplus challenge to both the anaesthetist and the surgeon. Collective decision-making tailored to the patient and close monitoring during the postoperative phase would result in good outcomes.

## Highlights

• Tracheobronchial injuries are uncommon complications during oesophagectomies.
• Various techniques exist for primary tracheobronchial repair.
• Muscle flaps and pericardial and pleural tissue are used for this purpose.
• Our patient had a successful repair with prolene and local transposition muscle flap using prevertebral muscles.
• Continuous monitoring for air leaks and mediastinitis is mandatory during intensive care.

## 1 Introduction

Mckeown oesophagectomy is a three-stage surgery performed primarily for mid-oesophageal cancers [1]. The anastomosis is performed in the cervical region with mobilization of oesophagus. An iatrogenic tracheal injury could occur during the dissection in the neck or thorax, both needing primary repair to avert life-threatening intraoperative and postoperative complications (such as air leaks, local and mediastinal infection), and prolonged hospital stay [2]. The Incidence of tracheal injuries is relatively low, quoted to be around 1–10 % [3], the majority associated with the posterior membranous portion of the trachea. The repair could be done using flaps (intercostal, pericardial) or simple approximation, the former being the preferred technique [2], [3], [4]. This case report discusses the management and outcome of an iatrogenic tracheal injury during the cervical dissection of a Mckeown oesophagectomy, managed with primary closure with prolene and prevertebral muscle flap and the postoperative course until 2 years. The paper is reported in line with SCARE criteria [5].

## 2 Case description

**Fig. 1:**
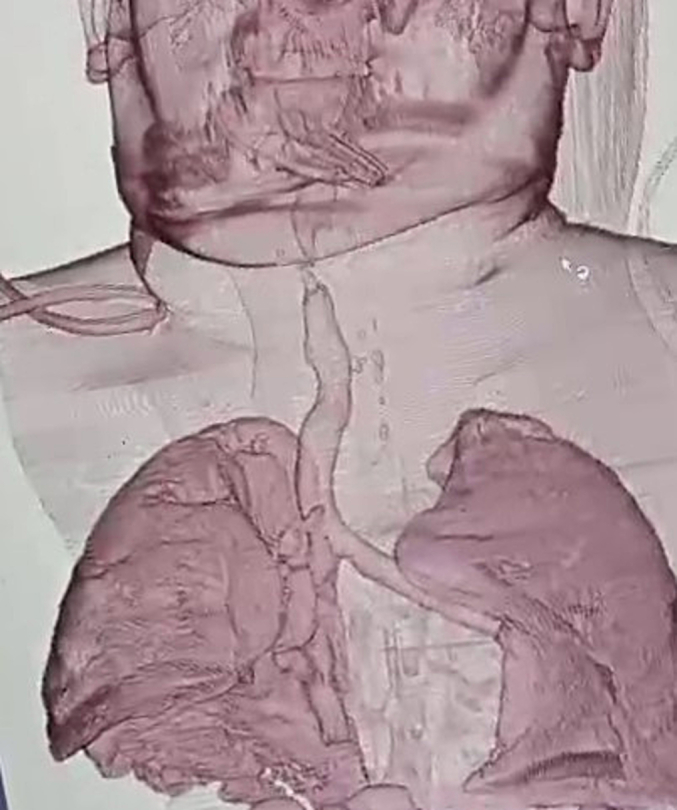
3D-reconstructed CT of the airway 2-years postop displaying no tracheal stenosis.

A 65-year-old South Asian male, a fisherman by profession (65 kg, BMI – 21 kg m^−2^) was planned for a Mckeown oesophagectomy for a moderately differentiated, mid-oesophageal squamous cell carcinoma staged as T3N2M0, in a District General Hospital in Sri Lanka. His smoking history revealed 20-pack years. In the preoperative contrast-enhanced computed tomography (CT), the tumour length was 4.2 cm. No local visceral organ or distant metastasis was noted. He received neo-adjuvant chemo radiotherapy. Chemotherapy was provided according to FOLFOX regimen. Following 03 cycles, the patient became unwell and refused further chemotherapy. RECIST criteria (response evaluation criteria in solid tumours) could not be applied fully due to the limited availability of CT facilities. Post-neoadjuvant endoscopy revealed visible tumour regression. His lung functions suggested a moderate obstruction (FEV1/FVC < 70 %, FEV1–40 %). The rest of the medical, surgical, and family history was insignificant. Prehabilitation was carried out with a high protein diet (serum protein-51 g/L) through jejunostomy, chest physiotherapy, and cessation of smoking. Three-stage Mckeown oesophagectomy was carried out 4-weeks post-chemotherapy with a thoracic epidural, invasive monitoring, and a double-lumen ETT (DLT) facilitating right thoracotomy. The thoracic and abdominal dissections and tumour mobilization were carried out without difficulty. During the cervical dissection, iatrogenic injury to the posterior membranous part of the trachea was observed while dissecting fibrous bands. It was a 2-cm laceration around the C7–8 level. The upper border of the DLT tracheal cuff was visible across the lower margin of the laceration. There was a leak of anaesthetic gases through the defect. The DLT was gently advanced distally with the use of video bronchoscope until the leak was no longer heard. Due to an unavailability of an oncosurgeon or an ENT surgeon at our institution, a collaborative decision was taken by the two-experienced surgeons to carry out primary closure of the defect by interrupted sutures with 2.0 prolene and local transposition muscle flap using prevertebral muscles. The defect was repaired carefully. A suction tube was advanced through the tracheal lumen of the DLT, tracheal cuff was deflated and gentle manual breaths were provided with the adjustable pressure limiting valve set at 20-cmH_2_O pressure. Instillation of 0.9 % saline around the repair did not reveal any air leaks. Subsequently, the cervical anastomosis was completed with meticulous dissection. Ryles tube was used throughout the surgery and used as an aid to identify the oesophagus during cervical dissection. DLT was replaced with a size 7.5 mm single lumen ETT under direct vision and the use of video-bronchoscope to avoid any disruption of the tracheal repair site by the ETT or inflated cuff. The right lung was inflated with an intercostal drain in-situ. The total operative time was 6 h and 45 min. At the completion of the procedure, the patient was admitted to intensive care with the ETT and sedated and ventilated for 48 h for the tissue oedema to settle and aid in healing the repair site. Continuous bubbling was noticed from the right intercostal drain. The chest x-ray revealed a small pneumothorax on the right lung. Hemodynamic parameters were stable and no mediastinal shift was noted in sequential X-rays. There was no subcutaneous emphysema in the neck suggesting disruption of the tracheal repair. On postoperative day 2, he was extubated under remifentanil cover which facilitated smooth extubation. Routine post-operative care was provided with gentle chest physiotherapy. The pneumothorax and the bubbling completely resolved with full expansion of the lung and the intercostal drainage was removed on day 3. On day 4, he was discharged to the ward HDU and home on day 07. The pathological staging was pT3N2M0. Postoperative chemotherapy was continued. He was followed up regularly at the outpatient clinic. At 2 years, he continued to abstain from smoking. He had no recurrence of the disease or symptoms suggestive of tracheal stenosis (Fig. 1).

## 3 Discussion

Tracheobronchial injury during oesophagectomy is uncommon [3], [4], [6]. Trans hiatal oesophagectomy which adopts blind dissection of the thoracic oesophagus is associated with majority of the injuries [7]. The incidence related to thoracoscopic techniques is low, however, statistically significant recent data are not available [8]. Other risk factors for tracheobronchial injuries are multiple. These include neoadjuvant chemo-radiotherapy, advanced tumour around mid-oesophagus, age > 65 years, and female sex [2], [3], [4], [8]. Procedure-related causes such as direct surgical injury and DLT insertions are also implicated in airway injuries [8], [9].

Injuries in the tracheobronchial tree might present in different ways. A direct surgical injury might be immediately visible as in our case. In other cases, either intraoperative or postoperative air leaks manifesting as subcutaneous emphysema, pneumothoraces, or continuous bubbling of intercostal drainage might point to the diagnosis [10]. Continuous bubbling of intercostal drainage in our patient was attributed to secondary pneumothorax subsequent to inadequate re-expansion of the lung (and insertion of a single lumen ETT) after capnothorax, in the absence of cervical subcutaneous emphysema or sepsis.

Once diagnosed, the decision needs to be taken on the need for repair. There exists the controversy of balancing out increased complications of primary repair and conservative management [11]. The need for thoracotomy in more distal injuries and subsequent post-operative sequela have contributed to increased mortality following surgically managed airway injuries in a few studies [11], some studies stating a mortality of 2.2 % [13]. The modes vary depending on the site, surgical expertise, and the extent of the injury [8], [11], [12]. It is suggested that injuries extending >2 cm, injuries which are detected intraoperatively, full-layer tracheal injuries of the posterior membranous portion, and injuries resultant from emergency intubation warrant primary repair [11], [14].

The surgical repair can be performed using tissue flaps of intercostal or pectoral muscle or pericardial or pleural origin or gastric tube with its omentum [15]. The primary repair might be at risk of postoperative radiotherapy [15]. Polytetrafluoroethylene, and repair with sutures and sealants are such described methods [8], [13]. There are recent reports of successful tracheobronchial repairs performed via robotic surgery [8]. After an airway injury, the patient can be extubated depending on the hemodynamics and the assumed stability of the repair. While it has been shown that the incidence of airway injuries during oesophagectomy varies inversely with the experience of the surgeons [8], faith in the primary repair would depend similarly on surgical expertise and experience. In our case, the operative time was extended with the repair of tracheal injury and the operating (general) surgeons had minimal experience in tracheobronchial repair. Thus it was collectively decided to ventilate the patient in the intensive care to rest the primary repair and to allow the airway oedema to subside. It is pertinent to closely monitor patients following airway injuries to detect persistent or new onset air leaks, postoperative pulmonary complications, and in worst-case scenarios, the development of mediastinitis. In the long term, patients might develop tracheal stenosis.

## 4 Conclusion

Tracheobronchial injuries during oesophagectomies are uncommon yet could lead to a complicated post-operative course immediately and long term. Early detection is paramount. Controversies exist on the ideal management pathway, either to proceed with conservative observation or primary repair. Whenever doubts exist, the opinion of surgeons experienced on the matter should be sought. Vigilance during postoperative intensive care specially for sustained air leaks and mediastinal infection is as important in any form of management. Novel techniques which include robotic surgeries are coming up which might further improve procedure-related morbidity.

## Consent

Written informed consent was obtained from the patient for the publication of this case report and accompanying images. A copy of the written consent is available for review by the Editor-in-Chief of this journal on request.

## Provenance and peer review

Not commissioned, externally peer-reviewed.

## Ethical approval

Our institution does not require ethical approval for reporting individual cases or case series.

## Funding

This research did not receive any specific grant from funding agencies in the public, commercial, or not-for-profit sectors.

## Guarantor

Dr. B.M. Munasinghe (Corresponding author).

## Research registration number

Not applicable.

## CRediT authorship contribution statement

Clinical management of the patient, concept, consent - BM, CK literature review, drafting of the initial and final manuscript, approval of the final manuscript - All authors.

## Conflicts of interest

None declared.
